# The Xyloglucan Galactosyltransferase EMT3 Regulates Diurnal Flowering Time by Modulating Lodicule Cell Wall Properties in Rice

**DOI:** 10.1111/pbi.70446

**Published:** 2025-11-16

**Authors:** Peizhou Xu, Maosen Ma, Kangxi Du, Tingkai Wu, Zhuchen Yao, Yuejiao Yin, Jian Wang, Xinhuan Liu, Zhen Zhang, Aiping Zhan, Changhui Sun, Duo Xia, Hai Zhou, Ming Luo, Xianjun Wu, Hao Zhou

**Affiliations:** ^1^ State Key Laboratory of Crop Gene Exploration and Utilization in Southwest China, Rice Research Institute Sichuan Agricultural University Chengdu China; ^2^ State Key Laboratory for Tropical Crops Breeding, Rubber Research Institute Chinese Academy of Tropical Agricultural Sciences Sanya China; ^3^ Guangdong Laboratory for Lingnan Modern Agriculture, State Key Laboratory for Conservation and Utilization of Subtropical Agro‐Bioresources College of Life Sciences, South China Agricultural University Guangzhou China; ^4^ Commonwealth Scientific and Industrial Research Organisation (CSIRO) Canberra Australia

**Keywords:** cell wall, floret opening time, hybrid rice, lodicule, xyloglucan galactosyltransferase

Seed production yield is a critical bottleneck limiting the large‐scale adoption of hybrid rice technology. Hybrid seed yield mainly depends on the outcrossing traits of the male sterile line, which can be broadly divided into two categories: floret‐opening traits and stigma‐related traits (Zhou et al. 2017). Among them, diurnal flowering time and stigma exsertion rate are the most decisive factors affecting outcrossing efficiency. However, asynchronous flowering, especially when the male sterile blooms later than the restorer in *indica*‐*japonica* hybrids, causes poor pollen reception and reduced seed yield. To date, only a few genes regulating diurnal flowering time in rice have been cloned (Gou et al. 2024; Wang et al. 2022; Xu et al. 2022), and the underlying genetic basis and molecular mechanisms remain poorly understood.

To identify new regulators of diurnal flowering time, we isolated an early‐ flowering mutant, *early morning flowering time 3 (emt3)*, from an EMS mutagenized population of Yixiang 1B (YX1B). Compared with its wild‐type (WT) YX1B, *emt3* initiated flowering about two hours earlier (Figure 1A,B; Figure S1A–C). Lodicules, located between the lemma and palea, are pivotal floral organs that regulate floret opening and closure in rice (Zhang et al. 2025). The lodicules in *emt3* were significantly larger than those in WT, both before and during anthesis (Figure 1C; Figure S1A). Transmission electron microscopy further revealed that the lodicule cell walls in *emt3* were significantly thinner than those in WT (Figure 1D; Figure S1B). Through water‐absorption experiments, we found that the lodicules of *emt3* absorbed water more rapidly and expanded faster than those of the WT (Figure S2). Thus, we hypothesize that these structural changes in *emt3* facilitate water uptake and expanding, thereby accelerating floret opening early.

**FIGURE 1:**
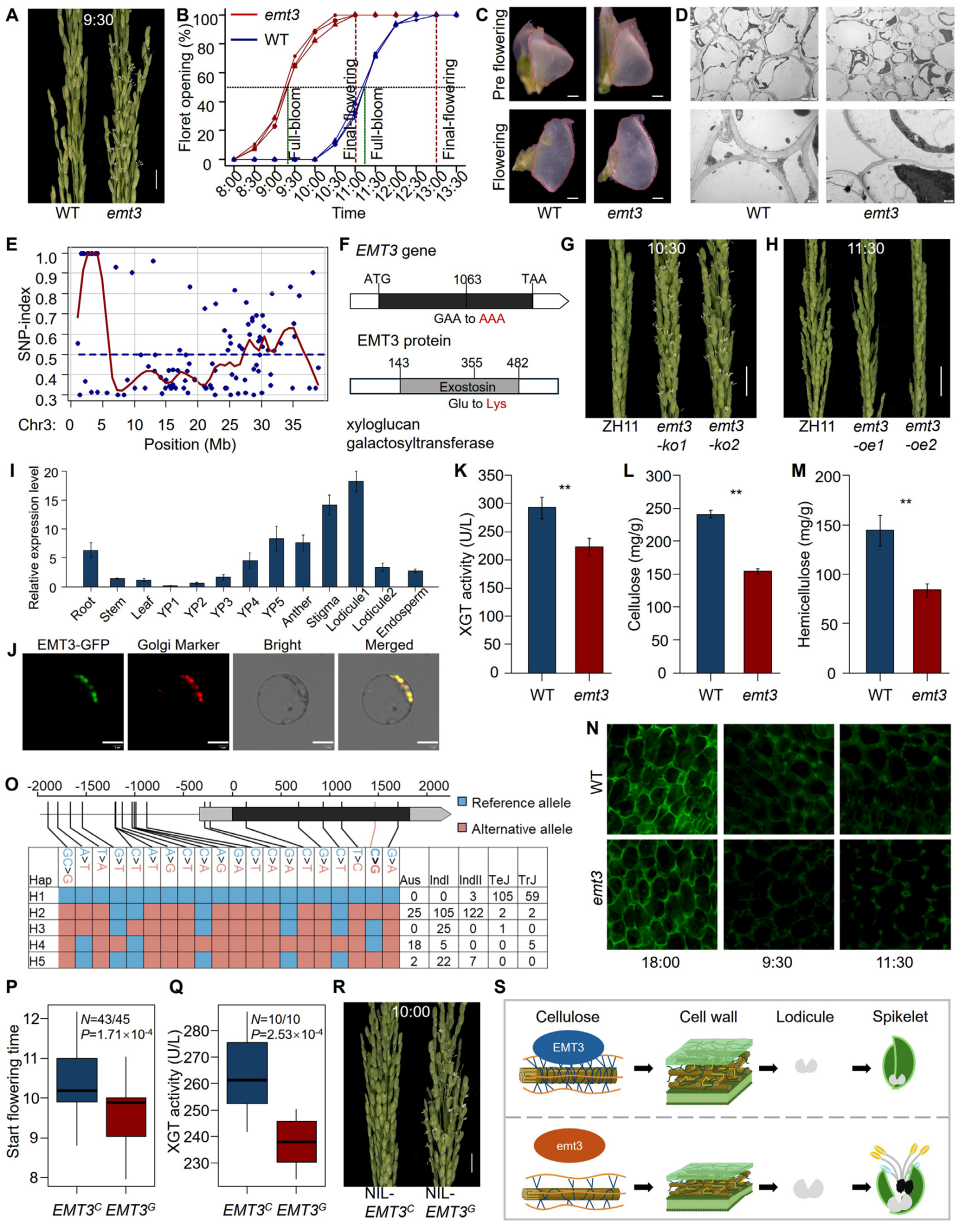
Gene cloning, functional analysis, and breeding potential evaluation of *emt3*. (A) Phenotype comparison showing earlier flowering in *emt3* versus WT. Bar = 2 cm. (B) Proportion of open florets over time. (C) Comparison of lodicule size in WT and *emt3* before (top) and after (bottom) flowering. Bar = 0.1 mm. (D) Lodicule cell morphology and cell wall thickness in WT and *emt3*. Bar = 5 μm and 1 μm. (E) MutMap analysis. (F) Gene structure and encoded protein domains *EMT3*. (G, H) Flowering phenotypes of *EMT3* knockout (*emt3‐ko*) mutants (G) and overexpression (*emt3‐oe*) lines (H) compared to ZH11. Bar = 2 cm. (I) Relative expression levels of *EMT3* across various rice tissues. (J) Subcellular localization of *EMT3* protein. Bar =10 μm. (K–M) XGT enzyme activity (K), cellulose content (L), and hemicellulose content (M) in lodicules of *emt3* compared to WT. Significance was determined by two‐tailed *t*‐test; ** indicates *p* < 0.01. (N) Immunofluorescence analysis of xyloglucan using LM15 antibody in lodicule cells. Bar = 10 μm. (O) Haplotype analysis of the *EMT3* gene. (P, Q) Comparison of flowering time (P) and XGT activity (Q) among rice accessions with different *EMT3* genotypes. Significance was evaluated by one‐way ANOVA. (R) Flowering time of near‐isogenic lines carrying two different *EMT3* alleles. Bar = 2 cm. (S) Schematic model illustrating the mechanism by which EMT3 regulates diurnal flowering time in rice.

For genetic mapping of the causal gene for *emt3*, Bulked segregant analysis (MutMap) identified a G‐to‐A single‐nucleotide variant causing a glutamate‐to‐lysine substitution (Figure 1E,F; Table S1). This mutation is located in *LOC_Os03g05110*, which is annotated as a xyloglucan galactosyltransferase (XGT) with high homology to *AtMUR3* in Arabidopsis (Figure S4). Mutations in *AtGT18*, a homologue of *AtMUR3* in Arabidopsis, reduce cellulose and hemicellulose content in the cell wall and cause a prostrate growth phenotype (Li et al. 2004). We therefore considered *LOC_Os03g05110* as the candidate gene for *EMT3*. Knockout and overexpression of *EMT3* in Zhonghua 11 (ZH11) confirmed its functional role: loss of function accelerated flowering, while overexpression delayed flowering (Figure 1G,H; Figure S5). Thus, *LOC_Os03g05110* was validated as *EMT3*, a regulator of diurnal flowering time in rice.

Both qRT‐PCR and GUS staining showed that *EMT3* is constitutively expressed, with enriched expression in young panicles, stigmas, and pre‐flowering lodicules (Figure 1I; Figure S6A). Subcellular localization confirmed that EMT3 resides in the Golgi apparatus (Figure 1J; Figure S6B), consistent with its predicted role in xyloglucan side‐chain synthesis (Julian and Zabotina 2022). Enzyme assays showed significantly reduced XGT activity in *emt3* lodicules (Figure 1K), accompanied by largely decreased cellulose and hemicellulose content (Figure 1L,M). Using immunofluorescence with the LM15 monoclonal antibody, which specifically recognises the XXXG oligosaccharide epitope of xyloglucan, we observed lower fluorescence in *emt3* lodicule cells compared to WT both 1 day before and during flowering (Figure 1N; Figure S7). These findings demonstrate that the *emt3* mutation impairs xyloglucan synthesis with reduced cellulose and hemicellulose, leading to thinner and more elastic cell walls that absorb water readily, thereby accelerating lodicule swelling and floret opening.

To assess the breeding potential of *emt3*, we compared the agronomic traits of WT with *emt3* plants. The *emt3* mutant exhibited a significant reduction in plant height (Figure S3). However, key yield components showed no significant differences, ultimately resulting in no measurable reduction in single‐plant yield. These results demonstrate that emt3 is a promising target for optimising flowering time in rice without compromising yield.

To further investigate the natural variation of the *EMT3* gene, we performed a haplotype analysis using a diverse panel of 533 cultivated rice accessions, which classified the *EMT3* into five major haplotypes: H1 was enriched in *japonica* (TeJ, TrJ), whereas H2‐H5 predominated in *indica* (Aus, IndI, IndII) (Figure 1O). A key C‐to‐G SNP at position 1460 caused a proline‐to‐arginine substitution at position 487 of the encoded protein, showing clear *indica*‐*japonica* differentiation (Figure 1O). The accessions carrying the *EMT3* ^*G*^ allele exhibited lower XGT activity and earlier flowering time than those with the *EMT3* ^*C*^ allele (Figure 1P,Q). We developed near‐isogenic lines (NILs) by introgressing the *japonica EMT3* ^*C*^ allele into the *indica* II‐32B background. NIL‐*EMT3* ^*C*^ flowered approximately 0.5 h later than NIL‐*EMT3* ^*G*^ (Figure 1R; Figure S8), suggesting that cumulative effects of multiple weak alleles contribute to flowering time divergence between *indica* and *japonica*.

## Author Contributions

Investigation: P.X., T.W. and M.M.; formal analysis: K.D., H.Z., D.X. and J.W.; resources: Z.Z., X.L. and A.Z.; visualisation: M.M., T.W. and H.Z.; writing: P.X. and H.Z.; supervision: M.L., C.S., H.Z. and X.W.; funding acquisition: P.X., H.Z. and X.W.; field management: J.W.

## Supporting information


**Data S1:** Supporting Information.

## Data Availability

The data that supports the findings of this study is available in the Supporting Information of this article.
