# Supplementary material for: The Xyloglucan Galactosyltransferase EMT3 Regulates Diurnal Flowering Time by Modulating Lodicule Cell Wall Properties in Rice
Source: Plant Biotechnol J. 2025 Nov 16;24(4):2015–7. doi: 10.1111/pbi.70446 (PMC13140295; doi:10.1111/pbi.70446)
Supplement: Supplementary file 1 — Data S1: Supporting Information. [file PBI-24-2015-s001.docx]

**Supplemental Data**

**The xyloglucan galactosyltransferase EMT3 regulates diurnal flowering time by modulating lodicule cell wall properties in rice**

Peizhou Xu^1#^, Maosen Ma^1#^, Kangxi Du^1#^, Tingkai Wu^1,2#^, Zhuchen Yao^1^, Yuejiao Yin^1^, Jian Wang^1^, Xinhuan Liu^1^, Zhen Zhang^1^, Aiping Zhan^1^, Changhui Sun^1^, Duo Xia^1^, Hai Zhou^3^, Ming Luo^4^, Xianjun Wu^1^*, Hao Zhou^1^*

1 State Key Laboratory of Crop Gene Exploration and Utilization in Southwest China, Rice Research Institute, Sichuan Agricultural University, Chengdu, 611130, China;

2 State Key Laboratory for Tropical Crops Breeding, Rubber Research Institute, Chinese Academy of Tropical Agricultural Sciences, Sanya, China

3 Guangdong Laboratory for Lingnan Modern Agriculture, State Key Laboratory for Conservation and Utilization of Subtropical Agro-bioresources, College of Life Sciences, South China Agricultural University, Guangzhou 510642, China

4 Commonwealth Scientific and Industrial Research Organisation (CSIRO), Australia

# Authors contributed equally

***For correspondence:** [zhouhao666@foxmail.com](mailto:zhouhao666@foxmail.com) (HZ); [wuxjsau@126.com](mailto:wuxjsau@126.com) (XW)

**Keywords:** hybrid rice, floret opening time, lodicule, cell wall, Xyloglucan galactosyltransferase

**Supplemental materials and methods, Figures S1–5, and Tables S1-2**.

**Materials and methods**

**Plant Materials and Cultivation**

The *emt3* mutant was identified from an EMS-mutagenized population of rice (O. *sativa* L. ssp. *indica*, Yixiang 1B). CRISPR/cas9-mediated knockout (*emt3-ko)* lines were generated in Zhonghua 11 (*O. sativa* L. ssp. *japonica*). Overexpression (*emt3-oe)* lines were created by introducing the *EMT3* coding sequence under the control of CaMV 35S promoter. A panel of 533 diverse cultivated rice varieties for haplotype analysis was kindly provided by prof. Gongwei Wang (Huazhong Agricultural University, Wuhan, China).

To construct the EMT3 near-isogenic lines (NILs), II-32B (*O sativa* L. ssp. *indica*) was crossed with Buphopa (*O sativa* L. ssp. *japonica*) to produce F_1_ progeny, which were then backcrossed three times with II-32B to yield the BC_4_F_1_ generation. NIL-EMT3^C^ and NIL-EMT3^G^ were selected from the segregating BC_4_F_2_ population. All plants were cultivated in the field of Sichuan Agricultural University, (Chengdu, China) or Nanfan Base in Lingshui (Sanya, China).

Three replications of the parents and F1 populations were grown in the normal rice-growing season at three locations at Ezhou in Wuhan. Seeds were planted in a seedbed in mid-May 2014, and approximately 30 plants of each variety/hybrid were transplanted to the field in mid-June. The planting density was 16.5 cm between plants in rows spaced 26 cm apart. Field management, including irrigation, fertilizer application and pest control, followed normal agricultural practices. Accessions were grown in a randomized block design, and the grains were harvested when fully ripe. Heading date, plant height, panicle length and panicle number were recorded in the field and grain-related traits, flower number, seed setting rate, grain number, grain length, grain width and grain weight, and were measured utilizing a highthroughput phenotyping facility (Yang et al., 2014).

**Evaluation of Flowering Time and Agronomic Traits**

The time-course statistics of floret opening ratio were conducted as follows: The count began when the earliest flowering materials (e.g., *emt3* compared to WT) initiated opening of their first floret. The number of opened florets from three individual plants per genotype was recorded at 10-minute intervals. The counting concluded when no new florets opened on the later-flowering materials for the day. The total number of florets opened per line on that day served as the denominator, and the number of florets opened by each time point (and all prior points) served as the numerator. The percentage of florets opened at each interval was calculated and plotted as a line graph.

The starting flowering time, full-bloom time, and final flowering time were defined as the time of the first floret opening, the time when 50% of florets had opened, and the time when 100% of florets had opened, respectively. The starting flowering time was used to investigate the association between flowering time and environmental conditions (temperature and humidity). Temperature and humidity data were obtained from a field weather station, which recorded ambient temperature, humidity, and dew point every 30 minutes. For simplified analysis, the average temperature and humidity during the morning period (6:00 AM to 12:00 PM) each day were used for correlation analysis with the flowering times of the different genotypes.

For the investigation of agronomic traits, WT and *emt3* were cultivated with three replicates at the Huimin Experimental Base of Sichuan Agricultural University in Chengdu, Sichuan Province, during the normal rice growing season. Seeds were sown in seedling beds in mid-May 2023 and transplanted to the field in mid-June. The planting density was set at 26 cm between rows and 16.5 cm between hills. Field management practices, including irrigation, fertilization, and pest control, followed conventional agricultural protocols. Grains were harvested at full maturity. Plant height, panicle length, and panicle number were recorded directly in the field, while grain-related traits (including floret number per panicle, seed setting rate, grain number per panicle, grain length, grain width, and grain weight) were measured using a high-throughput phenotyping platform(Yang *et al.*, 2014).

**Genetic analysis and gene mapping**

For MutMap analysis (Abe *et al.*, 2012), a BC_1_F_2_ segregation population was generated by backcrossing *emt3* with wild type (WT) YX1B. Thirty mutant plants with early floret opening were pooled as the “mut” group and 30 WT-like plants were pooled as the “WT” group. The DNA from each group was extracted, combined and subjected to high throughput sequencing in Oebiotech company (Shanghai, China). The clean reads of both groups were aligned to the Nipponbare reference genome (MSU version 7.0) using BWA software, and variants were identified using SAMtools (Li *et al.*, 2009) and GATK software (Md *et al.*, 2019).

**Cytological analysis**

High-contrast imaging of lodicule structures at maximum opening angle was performed using a ZEISS Xradia 510 Versa for WT and *emt3*, respectively with the following conditions: voxel resolution 4.5 μm, field of view 1.5×1.5 mm, voltage/power 50/4, and scanning time 0.9 s.

Transmission electron microscopy (TEM) was conducted to examine lodicule cell structure between WT and *emt3* at glume pre-opening and opening stages. The samples were prefixed with 3% glutaraldehyde, post-fixed with 1% osmium tetroxide, and dehydrated in a graded acetone series (30% - 100%). The tissues were filtered with acetone/epoxy resin (3:1, 1:1, 1), embedded, polymerized by heating and sectioned into ultra-thin slices for double staining with lead citrate and uranium acetate and observing with a Hitachi H-600IV TEM.

For the water-absorption experiments, we collected the lodicules from WT and *emt3* florets at 6 p.m., one day before anthesis for examination under a microscope. A 3 µL droplet of distilled water was applied to each sample simultaneously for imaging every 10 seconds to monitor water uptake dynamics.

**Vector construction and plant transformation**

DNA fragments were amplified and cloned into binary vectors for transformation (primer sequences listed in Table S2).

CRISPR/Cas9 knockout (KO-EMT3): 23 bp targets with NGG PAMs at the *EMT3* C-terminal were selected. Twenty bp targets were cloned into intermediate vectors (pER8-Cas9-U6 or pER8-Cas9-U3) and subsequently into pCXUN-Cas9. The constructs were independently introduced into ZH11.

Overexpression (OE-EMT3): *EMT3* cDNA from YX1B was cloned into pCAMBIA1300 under the CaMV 35S promoter (using BamH1 and Ecor1). The constructs were introduced into ZH11.

All the constructs were validated by sequencing in E. coli strain Trans 5α, introduced into *Agrobacterium tumefaciens* strain EHA105, and transferred into rice. The knockout genotypes were confirmed by sequencing a 200 bp flanking region around the 20 bp target site, and by amplifying a 750 bp Cas9 fragment. Homozygous edited lines were selected for flowering-time analysis.

**Expression analysis**

Total RNA was extracted using TRIzol (Invitrogen 15596-026, USA), and first-strand cDNA was synthesized with M-MLV reverse transcriptase (Invitrogen C28025-014, USA). qRT-PCR was performed on a QuantStudio6 Flex using SYBR Green (Thermo Fisher). Young panicles and endosperm samples were collected at different stages, with ≥30 grains per plant. Each sample was analyzed with 3–5 biological replicates and three technical replicates. Gene *UBQ* was used as the internal reference.

**Gene function and subcellular localization**

*EMT3* gene is annotated as a putative xyloglucan galactosyltransferase, homologous to KATAMARI1 (https://www.ricedata.cn/gene/). To clarify the protein function of *EMT3*, SMART (<http://smart.embl-heidelberg.de/>) analysis predicted one transmembrane helix (aa 29–51), two low-complexity regions (aa 56–73, 85–101), and an Exostosin conserved domain (aa 143–482).

For subcellular localization, *EMT3* was fused to GFP in the pCAMBIA1301S vector (Zhang *et al.*, 2011). EMT3-GFP and nuclear/cell membrane markers were transiently expressed in rice protoplasts and *Nicotiana benthamiana* leaves (Voinnet *et al.*, 2003). Fluorescence for proteins was visualized using a Leica TCS SP2 confocal microscope. All fluorescence assays were repeated ≥3 times.

**Immunofluorescence**

Immunofluorescence was performed as described by Wang et al. (2022). Paraffin sections were dewaxed in xylene, rehydrated through a graded ethanol series (100, 95, 90, 80, 60, and 30%), and subjected to antigen retrieval in 0.01 M sodium citrate buffer (pH 6.0) at 95 °C for 10–15 min. After blocking with 3% bovine serum albumin (BSA) for 30–60 min., the sections were incubated overnight at 4 °C with LM15 antibody (PlantProbes) in 3% BSA, followed by GFP-conjugated secondary antibody for 2 h at room temperature in the dark. After PBS washes, the sections were mounted in 10% glycerol and imaged with a confocal laser scanning microscope.

**Haplotype analysis.**

For haplotype analysis of *EMT3*, SNP data from the 2 kb promoter and 3.1 kb coding region were extracted from 533 diverse rice accessions (Zhao *et al.*, 2015). The variants with minor allele frequency (MAF) >0.03 were retained for haplotype analysis, which population structure was determined as described by Zhou *et al.* (2017).

**Primers.**

The primers used in this study are listed in Table 2

**Supplemental reference**

Abe, A., Kosugi, S., Yoshida, Kentaro, Natsume, S., Takagi, H., Kanzaki, H., et al. (2012) *Genome sequencing reveals agronomically important loci in rice using MutMap*. *Nat. Biotechnol.*, **30**, 174–178.

Li, H., Handsaker, B., Wysoker, A., Fennell, T., Ruan, J., Homer, N., et al. (2009) *The sequence alignment/map format and SAMtools*. *Bioinformatics*, **25**, 2078–2079.

Md, V., Misra, S., Li, H., and Aluru, S. (2019) *Efficient architecture-aware acceleration of BWA-MEM for multicore systems*. In: *Proceedings - 2019 IEEE 33rd International Parallel and Distributed Processing Symposium, IPDPS 2019* , pp. 314–324. IEEE.

Voinnet, O., Rivas, S., Mestre, P., and Baulcombe, D. (2003) *Retracted: An enhanced transient expression system in plants based on suppression of gene silencing by the p19 protein of tomato bushy stunt virus*. *plant J.*, **33**, 949–956.

Wang, M., Zhu, X., Peng, G., Liu, M., Zhang, S., Chen, M., et al. (2022) *Methylesterification of cell-wall pectin controls the diurnal flower-opening times in rice*. *Mol. Plant*, **15**, 956–972.

Yang, W., Guo, Z., Huang, C., Duan, L., Chen, G., Jiang, N., et al. (2014) *Combining high-throughput phenotyping and genome-wide association studies to reveal natural genetic variation in rice*. *Nat. Commun.*, **5**, 5087.

Zhang, Y., Su, J., Duan, S., Ao, Y., Dai, J., Liu, J., et al. (2011) *A highly efficient rice green tissue protoplast system for transient gene expression and studying light/chloroplast-related processes*. *Plant Methods*, **7**, 30.

Zhao, H., Yao, W., Ouyang, Y., Yang, W., Wang, G., Lian, X., et al. (2015) *RiceVarMap: a comprehensive database of rice genomic variations*. *Nucleic Acids Res.*, **43**, D1018-22.

Zhou, H., Li, P., Xie, W., Hussain, S., Li, Y., Xia, D., et al. (2017) *Genome-wide association analyses reveal the genetic basis of stigma exsertion in rice*. *Mol. Plant*, **10**, 634–644.


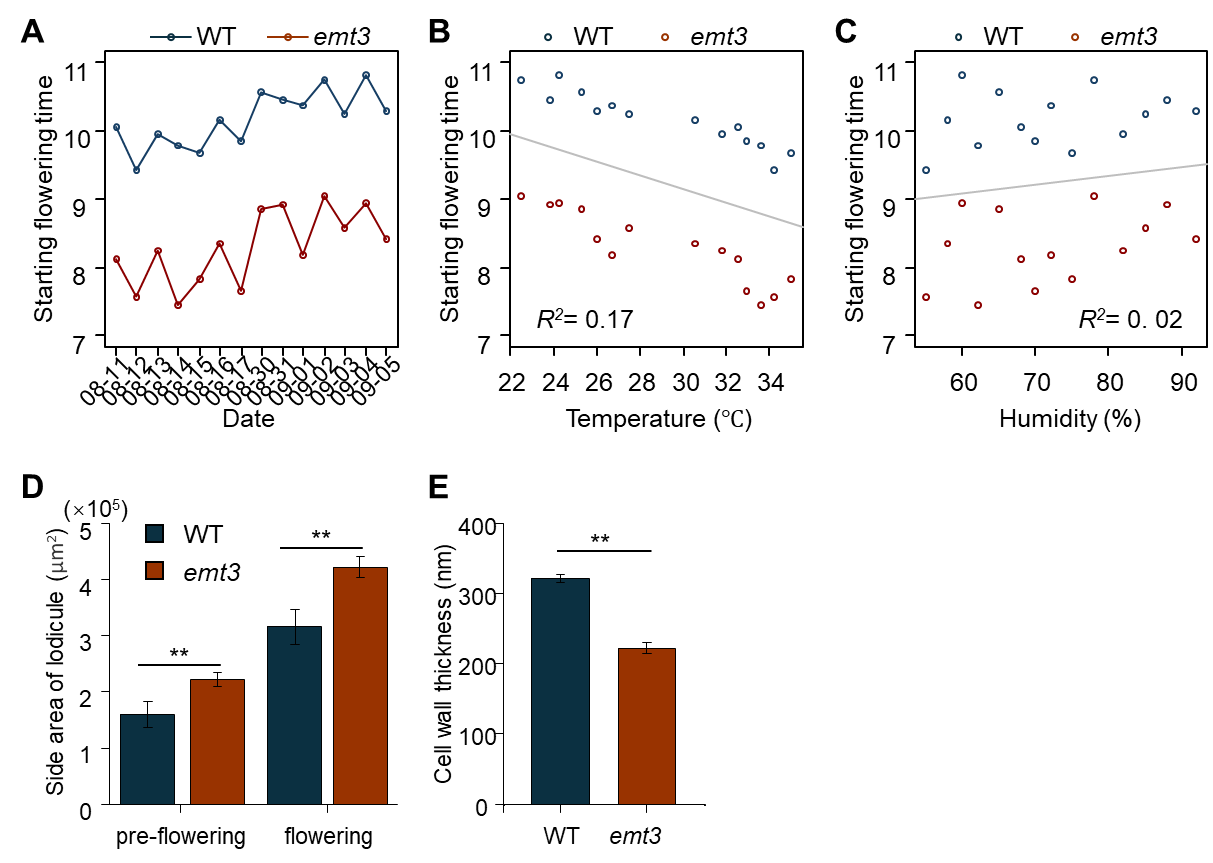


Figure S1. Differences in flowering time and lodicule morphology between WT and *emt3*. (A) Flowering onset time of WT and *emt3* observed on different dates. (B–C) Correlation between flowering time and environmental parameters: (B) temperature and (C) humidity in WT and *emt3*. (D-E) Comparison of lodicule side area (D) and cell wall thickness (E) in WT and *emt3*. Statistical significance ** at *P* < 0.01, two-tailed *t*-test.


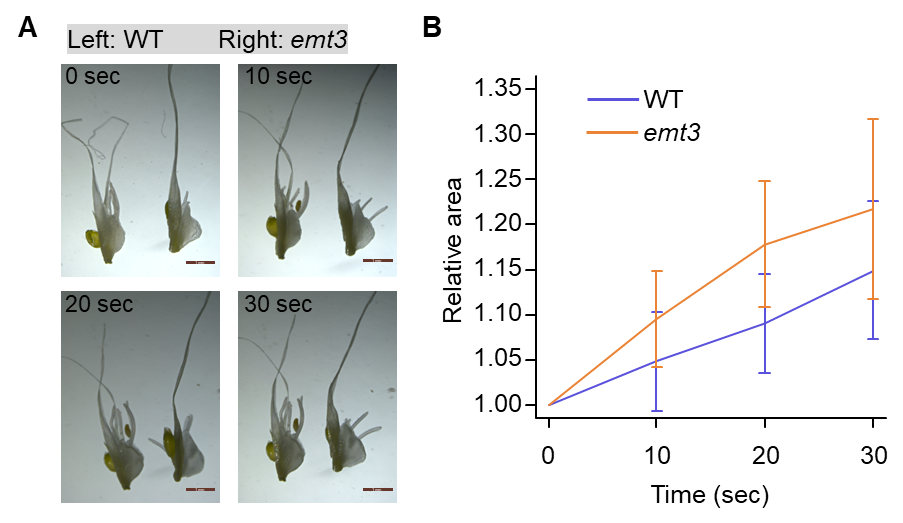


Figure S2. Comparison of lodicule water absorption between WT and *emt3* mutant. (A) Morphology changes in lodicules after water absorption. Scale bar = 1 mm. (B) Time-course of lodicule surface area expansion after water treatment.


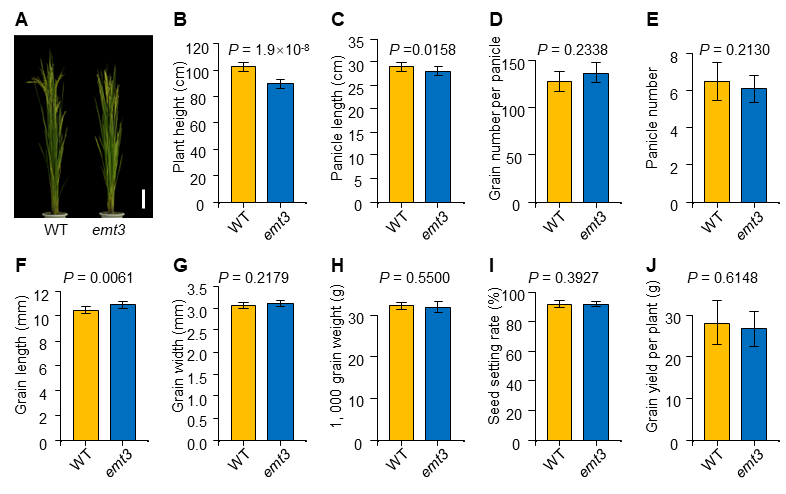


Figure S3. Comparison of agronomic traits between WT and *emt3* mutant. All *P*-values for significance are based on two-tailed *t*-tests.


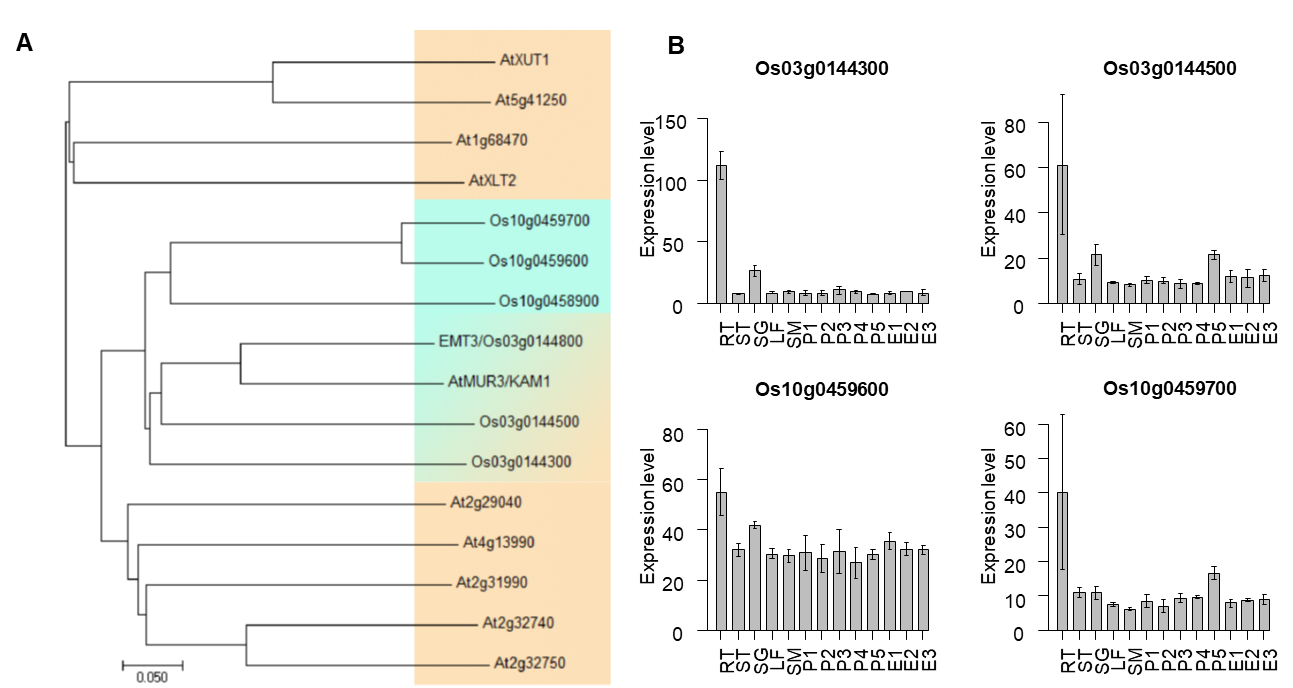


Figure S4. Phylogenetic tree analysis of *EMT3* homologous proteins in Arabidopsis thaliana and rice.


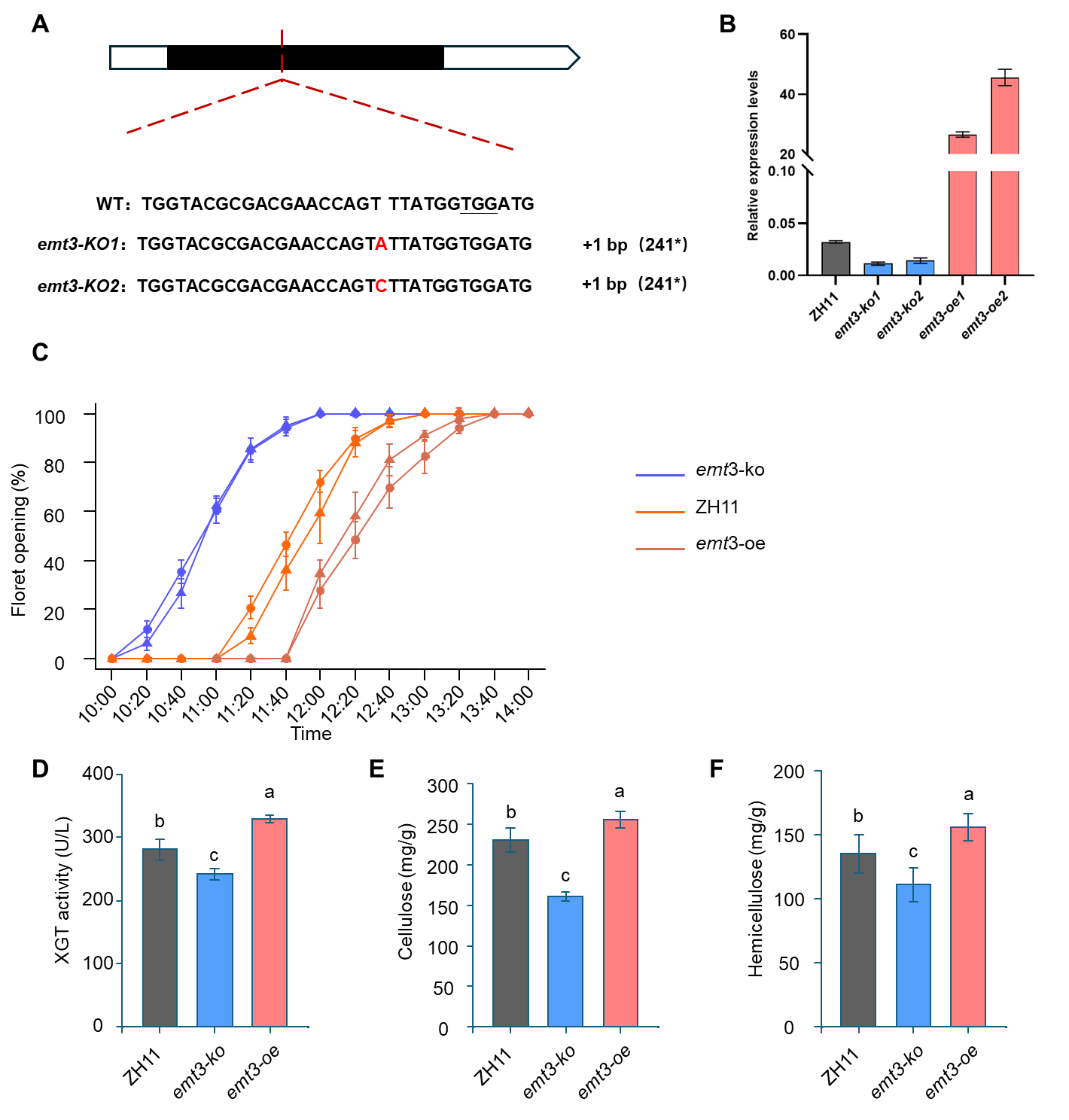


Figure S5. Phenotypes of *EMT3* knockout (*emt3-ko*) and overexpression (*emt3-oe*) lines. (A) Genotypes of *emt3* knockout mutants (*ko1* and *ko2*) generated by CRISPR/ Cas9-mediated gene editing. (B) Expression levels of *EMT3* in ZH11, *emt3-ko* and *emt3-oe* lines. (C) Proportion of open florets over time in ZH11, *emt3-ko* and *emt3-oe* lines. (D-F) Xyloglucan galactosyltransferase (XGT) activity (D), cellulose content (E) and hemicellulose content (F) in ZH11, *emt3-ko* and *emt3-oe* lines. Different lowercase letters indicate significant differences (*P* < 0.05) according to Duncan's multiple range test.


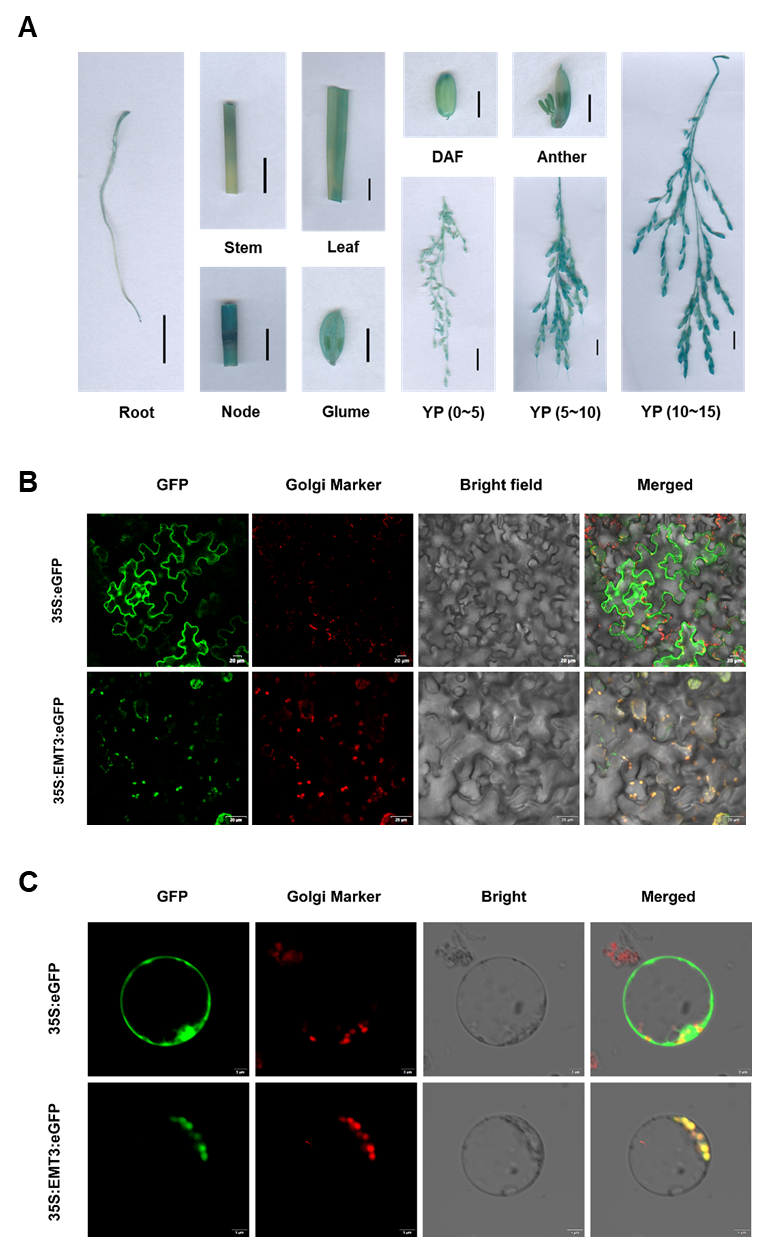


Figure S6. Expression Pattern of *EMT3* Gene and Subcellular Localization of EMT3 Protein.

(A) GUS staining in different tissues and developmental stages of EMT3-GUS transgenic plants. (B-C) Subcellular localization of EMT-GFP and golgi marker in tobacco leaf epidermal cells (B) and rice protoplasts (C).


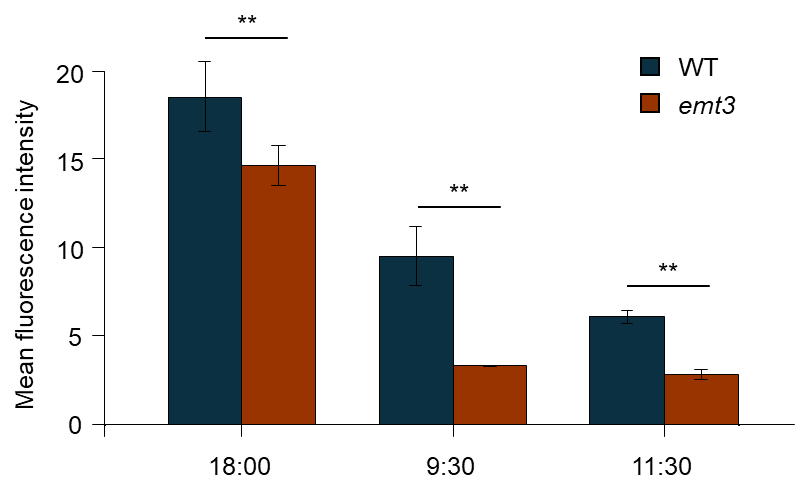


Figure S7. Immunofluorescence analysis of xyloglucan distribution using LM15 antibody in lodicule cells of WT and *emt3*. Statistical significance ** at *P* < 0.01, two-tailed *t*-test.


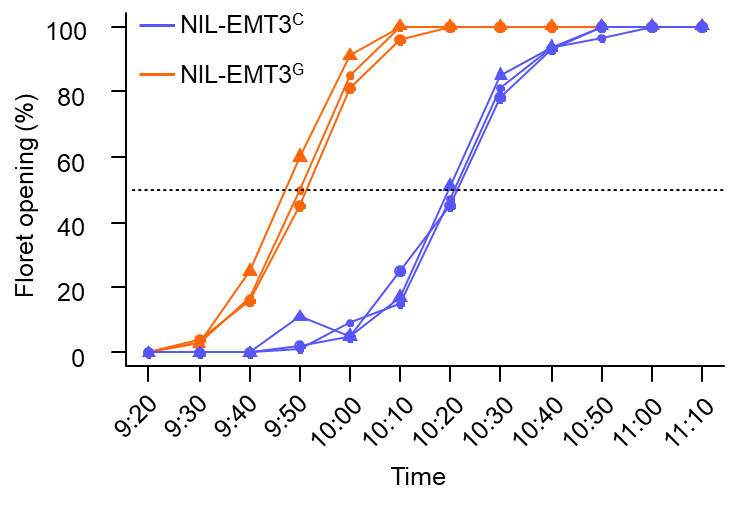


Figure S8. Percentage of floret opening of two *EMT3* alleles in maintainer II-32B background.

Table S1. Variations with SNP-index=1 on chromosome 3 in MutMap analysis

| Chr | Pos | Var | Type | Gene ID | Effect | Annotation |
| --- | --- | --- | --- | --- | --- | --- |
| 3 | 1575004 | G/A | upstream | Os03g0127900 | MODIFIER | transporter, Aproton antiporter-2 family |
| 3 | 1720315 | A/G | 3'UTR | Os03g0130500 | MODIFIER | EF hand family protein |
| 3 | 2488323 | G/A | missense | Os03g0144800 | MODERATE | xyloglucan galactosyltransferase |
| 3 | 2515434 | G/A | 3'UTR | Os03g0145300 | MODIFIER | expressed protein |
| 3 | 4109873 | G/A | upstream | Os03g0177900 | MODIFIER | elongation factor Tu |
| 3 | 5441370 | G/A | upstream | Os03g0203750 | MODIFIER | Non-protein coding transcript |

Table S2. Primers used in this study

| **Primer name** | **Sequence (5'–3')** | **Purpose** |
| --- | --- | --- |
| 1063-F | AGTCGGATTGGGGCAACA | Gene location |
| 1063-R | CGCTAGGGGAATGGCACT |  |
| *EMT3-KO-F* | CTCTGGACCAATATGTGCAAG |  |
| *EMT3-KO-R* | TACCTCGCCACATCAAACCC |  |
| *EMT3-OX-F* | atttggagaggacagggtaccATGTCTGCTATGAGGCGGCG | 35S::eGFP and GUS vector |
| *EMT3-OX-R* | ggtactagtgtcgactctagaGTTCTGACCTCTTTGTTCGTTTTTC |  |
| *ProEMT3-GUS-F* | tggctgcaggtcgacggatccCGAGAGAGTGATCACGATTTGGC |  |
| *ProEMT3-GUS-R* | aaaacgacggccagtgaattcGAAGAGGAAACCAGGTGGTGTG |  |
| UBQ-RT-F | AACCAGCTGAGGCCCAAGA | RT-qPCR |
| UBQ-RT-R | ACGATTGATTTAACCAGTCCATGA |  |
| *qEMT3-F* | GGCACAACATTTCGACGAGG |  |
| *qEMT3-R* | CAATCCGACTCTTCGTCCGT |  |
| gRT1: | TACGCGACGAACCAGTTTAgttttagagctagaaat | CRISPR/Cas9 |
| OsU6aT1: | TAAACTGGTTCGTCGCGTACggcagccaagccagca |  |
